# Venom complexity of *Bothrops atrox* (common lancehead) siblings

**DOI:** 10.1590/1678-9199-JVATITD-2020-0018

**Published:** 2020-10-12

**Authors:** Daniela Miki Hatakeyama, Lídia Jorge Tasima, Cesar Adolfo Bravo-Tobar, Caroline Serino-Silva, Alexandre Keiji Tashima, Caroline Fabri Bittencourt Rodrigues, Weslei da Silva Aguiar, Nathália da Costa Galizio, Eduardo Oliveira Venancio de Lima, Victor Koiti Kavazoi, Juan David Gutierrez-Marín, Iasmim Baptista de Farias, Sávio Stefanini Sant’Anna, Kathleen Fernandes Grego, Karen de Morais-Zani, Anita Mitico Tanaka-Azevedo

**Affiliations:** 1 Laboratory of Herpetology, Butantan Institute, São Paulo, SP, Brazil.; 2 Interinstitutional Graduate Program in Biotechnology (IPT, IBU and USP), University of São Paulo (USP), São Paulo, SP, Brazil.; 3 Department of Biochemistry, Federal University of São Paulo (Unifesp), São Paulo, SP, Brazil.

**Keywords:** Bothrops atrox, Snake venom, Individual variation, Envenomation

## Abstract

**Background:**

Variability in snake venoms is a well-studied phenomenon. However, sex-based variation of *Bothrops atrox* snake venom using siblings is poorly investigated. *Bothrops atrox* is responsible for the majority of snakebite accidents in the Brazilian Amazon region. Differences in the venom composition of *Bothrops* genus have been linked to several factors such as ontogeny, geographical distribution, prey preferences and sex. Thus, in the current study, venom samples of *Bothrops atrox* male and female siblings were analyzed in order to compare their biochemical and biological characteristics.

**Methods:**

Venoms were collected from five females and four males born from a snake captured from the wild in São Bento (Maranhão, Brazil), and kept in the Laboratory of Herpetology of Butantan Intitute. The venoms were analyzed individually and as a pool of each gender. The assays consisted in protein quantification, 1-DE, mass spectrometry, proteolytic, phospholipase A_2_, L-amino acid oxidase activities, minimum coagulant dose upon plasma, minimum hemorrhagic dose and lethal dose 50%.

**Results:**

Electrophoretic profiles of male’s and female’s venom pools were quite similar, with minor sex-based variation. Male venom showed higher LAAO, PLA_2_ and hemorrhagic activities, while female venom showed higher coagulant activity. On the other hand, the proteolytic activities did not show statistical differences between pools, although some individual variations were observed. Meanwhile, proteomic profile revealed 112 different protein compounds; of which 105 were common proteins of female’s and male’s venom pools and seven were unique to females. Despite individual variations, lethality of both pools showed similar values.

**Conclusion:**

Although differences between female and male venoms were observed, our results show that individual variations are significant even between siblings, highlighting that biological activities of venoms and its composition are influenced by other factors beyond gender.

## Background

Snakebite envenomation is considered a worldwide Category A neglected tropical disease and constitutes a public health problem in warmer regions of the developing world [1,2]. In Latin America, the family Viperidae is responsible for most of the registered snakebite accidents, and in Brazil, the genus *Bothrops* is responsible for 85% of the ophidian envenomation [1-5].

*Bothrops atrox* (common lancehead) is a pit viper species widely distributed in the northern region of South America [7-9] and its natural history is already well documented [10]. This generalist species occurs mostly in rainforests, but can also be found in disturbed areas. In relation to other *Bothrops* species, the common lancehead shows preference towards heavier preys [11]. Males are smaller than females and are more prone to higher mortality, considering the active foraging lifestyle of the species. In fact, *B. atrox* exhibits a dynamic use of its habitat, being known as one of the most active hunters of the *Bothrops* genus [9,^11^,^12^]. *B. atrox* venom causes mainly local damage, such as edema, hemorrhage and necrosis, apart from systemic effects, including blood coagulation disorders [13,14]. In lethal cases, hemorrhage leads to cardiovascular shock and acute renal failure secondary to acute tubular necrosis and occasionally glomerulonephritis [7,15]. These symptoms are the result of individual or synergistic action of different toxins that comprise the venom of snakes [16,17], such as phospholipases A_2_ (PLA_2_s), metalloproteinases (SVMPs), serine proteinases (SVSPs), L-amino acid oxidases (LAAOs), among others [1,18]. The knowledge about the composition and action of snake venoms allows us to understand the evolutionary processes in ophidians [19] and elucidate the mode of action of toxins and the demand for their antagonists [20]. In addition, as snake venoms are a rich source of bioactive compounds with pharmaceutical potential, they can represent an improvement in snakebite envenoming treatment, which can impact significantly on the victims symptoms and the quality and efficacy of antivenoms [21,22].

Individual variability is a well-established concept when referring to intraspecific variation of snake venom composition and/or its activities, and may be related to ontogeny [23-25], diet [26,27], seasonality [28], geographical location [29-31], gender [32-35], and captivity [22,36]. Within the *Bothrops* species, *B. jararaca* venom is the most studied one regarding gender differences [32,37], contrary from *B. atrox*, despite its high geographic distribution and epidemiological representation. In this context, the present study aims to compare, for the first time, the biochemical and biological characteristics of male and female venom of *B. atrox* siblings. Both genders were born in captivity and maintained under controlled conditions, in order to contribute to the knowledge of changes in venom characteristics according to sex, as well as the formulation of pharmacological tools for inhibiting the toxic effects of this venom.

## Methods

### Animals

*Mus musculus* (Swiss) male mice (18-22 g) were obtained from Butantan Institute animal house, had access to water and food *ad libitum* and were kept under a 12 h light/dark cycle. *B. atrox* specimens (5 females and 4 males over 11 years of age) (Additional file 1) were born from the same snake captured from the wild (São Bento, Maranhão, Brazil), and kept in the Laboratory of Herpetology of Butantan Institute under controlled conditions.

### Venoms

The venom was extracted from nine *B. atrox* snakes (5 females and 4 males born from the same mother), centrifuged for 15 min at 1700 × *g*, 4 ºC, to remove any scales or mucus, lyophilized, and stored at -20 ºC until use. Information regarding the snakes is available in Additional file 1. 

### Compositional analysis

**Protein quantification**

Protein concentration of pools (female and male) and individual venom samples was determined according to the Bradford method, using Bio-Rad Protein Assay reagent and bovine serum albumin (BSA) (Sigma) as standard [38]. These data were only used as a basis to other experiments.

**One-dimensional electrophoresis (1-DE)**

Electrophoretic analysis of pools and individual venom samples was performed using 30 µg of protein in the presence and absence of β-mercaptoethanol in 15% polyacrylamide gels [39]. The gels were stained with Coomassie Blue G according to the GE Healthcare protocol.

**Protein identification by mass spectrometry**

Identification of proteins was performed by LC-MS/MS in a Synapt G2 (Waters) coupled to the nanoAcquity UPLC chromatographic system (Waters) as previously described [40,41]. Briefly, samples of 100 μg of protein from each venom pool were incubated in 50 mM ammonium bicarbonate with 5 mM DTT (dithiothreitol) for 25 min at room temperature (RT), followed by addition of 14 mM IAA (iodoacetamide) and incubation in the dark for 30 min at RT. Finally, an incubation with 5 mM DTT for 15 min was performed. Calcium chloride (1 mM) and 1 µg of trypsin (Sigma) in 50 mM ammonium bicarbonate were added to each sample and incubated for 16 h at 37 °C. After incubation, the reaction was stopped with 5% TFA (0.5% final concentration). Aliquots of the resulting peptide mixtures (5 μg) were injected into a trap column packed with C18 (nanoAcquity trap Symmetry 180 μm × 20 mm) at 8 µL/min with phase A (0.1% formic acid. Peptides were then eluted onto an analytical C18 column (nanoAcquity BEH 75 μm × 200 mm, 1.7 m) at a flow rate of 275 nL/min, using a gradient of 7-35% of phase B (0.1% formic acid in acetonitrile) in 90 min. Data were acquired in the in data-independent mode UDMSE [42] in the m/z range of 50-2000 and in resolution mode. Collision energies were alternated between 4 eV and a ramp of 17-60 eV for precursor ion and fragment ions, respectively, using scan times of 1.25 s. The ESI source was operated in positive mode with a capillary voltage of 3.0 kV, block temperature of 70 °C, and cone voltage of 40 V. For lock mass correction, [Glu1]-Fibrinopeptide B solution (500 fmol/mL in 50% acetonitrile, 0.1% formic acid; Peptide 2.0) was infused through the reference sprayer at 500 nL/min and sampled for 0.5 s at each 60 s.

Raw data were processed in ProteinLynx Global Server 3.0.1 (Waters) by the Apex3D module using low energy threshold of 750 counts and elevated energy threshold of 50 counts. MS/MS spectra were submitted to searches a Serpentes database (downloaded from Uniprot in March 1^st^, 2019, 2608 reviewed sequences). The following search parameters were used: automatic fragment and peptide mass tolerances, carbamidomethylation of cysteines as fixed modification, oxidation of methionine, N-terminal acetylation, glutamine and asparagine deamidation as variable modifications, up to 2 missed cleavage sites were allowed for trypsin digestion. The following criteria were set for protein identification: a minimum of 1 fragment ion per peptide, 5 fragment ions per protein and 2 peptides per protein, and a maximum false discovery identification rate of 1%, estimated by a simultaneous search against a reversed database. Label-free quantitative assessments were based on the average intensities of the three most intense peptides of each identified protein [43]. Each pooled sample was analyzed in technical triplicate. Data of the spectra are available in Additional file 2.

### Enzymatic activities

**Caseinolytic activity**

Caseinolytic activity was determined as described [44] using azocasein (Merck) as substrate. Briefly, 85 µL of a 4.25 mg/mL azocasein solution were incubated with 10 µL of each venom (1 mg/mL), both diluted in 50 mM Tris-HCl buffer, pH 8.0. The reaction was stopped by adding 200 µL of 5% trichloroacetic acid (TCA). The samples were centrifuged at 1000 × g and 100 µL of the supernatant were homogenized with 100 µL of 0.5 M NaOH. The absorbance was measured at 450 nm in a SpectraMax i3 microplate reader (Molecular Devices). One unit of activity was determined as the amount of venom that induces an increase of 0.005 units of absorbance.

**Collagenolytic activity**

Collagenolytic activity over azocoll was determined according to Váchová and Moravcová [45] and modified by Antunes et al. [46]. Venoms (6.25 µg) were incubated with 50 µL of a 5 mg/mL azocoll (Sigma) solution, both diluted in Tyrode buffer (137 mM NaCl, 2.7 mM KCl, 3 mM NaH_2_PO_4_, 10 mM HEPES, 5.6 mM dextrose, 1 mM MgCl_2_, 2 mM CaCl_2_, pH 7.4) for 1 h in constant shake, at 37 °C. The samples were centrifuged for 3 min at 5000 × g and the absorbance of the supernatants (200 µL) was measured at 540 nm in a SpectraMax i3 microplate reader (Molecular Devices). One unit of activity was determined as the amount of venom that induces an increase of 0.003 units of absorbance.

**L-amino acid oxidase activity**

Pools and individual venom samples were analyzed by measuring the hydrogen peroxide generated during the oxidation of L-amino acids [47]. For this, 5 μg of the venom were added to the 90 µL reaction mixture containing 50 mM Tris-HCl, 250 mM L-methionine, pH 8.0, 2 mM o-phenylenediamine and 0.8 U/mL of horseradish peroxidase, and the mixture incubated at 37 ºC for 60 min. The reaction was stopped using 50 μL of 2 M H_2_SO_4_ and the absorbance measured on a spectrophotometer (SpectraMax i3, Molecular Devices) at 492 nm. Results were expressed as 1 μM of H_2_O_2_/minute/µg of venom.

**Phospholipase A_2_ activity**

The phospholipase A_2_ (PLA_2_) activity of pools and individual venom samples was determined based on the assay developed by Holzer and Mackessy [48] using the monodisperse synthetic substrate 4-nitro-3-octanoyloxy-benzoic acid (NOBA). Twenty µg of venom (dissolved in 0.85% NaCl), 20 µL of deionized water and 200 µL of 10mM Tris-HCl, 10 mM CaCl_2_, 100 mM NaCl, pH 8.0 were mixed in a 96 well microplate. Then, 20 µL of NOBA (4.16 mM in acetonitrile) was added in a final concentration of 0.32 mM. After incubating for 20 min at 37 ºC, the absorbance at 425 nm was recorded in a microplate reader (SpectraMax i3, Molecular Devices). A change of 0.1 absorbance unit at 425 nm was equivalent to 25.8 nmoles of chromophore release.

### Biological functions

**Coagulant activity**

The coagulant activity of the venom pools was assessed in citrated human plasma, according to Theakston and Reid [49]. Briefly, 100 μL of plasma were incubated at 37 °C for 60 s. After the incubation, 50 µL of various concentrations of venom samples were mixed and clotting times were measured in a coagulometer (MaxCoag, MEDMAX). The Minimum Coagulant Dose (MCD) was defined as the minimum amount of venom that induced coagulation of plasma in 60 s at 37 °C.

**Hemorrhagic activity**

The hemorrhagic activity was obtained by the determination of Minimum Hemorrhagic Dose (MHD). Groups of five male Swiss mice of 18-22 g were injected with 100 μL of several doses of venom pool samples, diluted in 0.89% NaCl, intradermally into the venter of the mice, and a control group received 100 μL of NaCl solution under identical conditions. After 3 h, the animals were euthanized in a CO_2_ chamber, the venter skin was removed, and the hemorrhagic areas were measured [50]. The MHD was defined as the amount of venom that produced hemorrhages with a mean diameter of 10 mm after 3 h [51].

**Median lethal dose (LD_50_)**

The LD_50_ of venom pool samples were determined by intraperitoneal injection in 18-22 g male Swiss mice with 500 μL of varying doses of venoms (66-381 µg/animal) in 0.89% NaCl. Five mice were used per group and the number of deaths occurring within 48 h after injection was recorded. The LD_50_ and 95% confidence intervals were calculated by Probit analysis [52].

### Immunorecognition by antibothropic serum

Individual venoms and pools (30 µL) were submitted to 1-DE (15%) under reducing conditions (as described in the section “One-dimensional electrophoresis (1-DE)”) and transferred to PVDF membranes (Bio-Rad) in a semi-dry system (Trans-Blot Turbo Transfer System, Bio-Rad) at 25 V for 35 min. As described by Harlow and Lane [53], the membranes were blocked with Tris-buffered-saline containing 5% fat free milk (TBS-milk) overnight at 4 °C. The membranes were incubated with 1:2,000 commercial antibothropic serum (batch 1305077, expiration date due to 2016) for 2 h at room temperature. After washing with TBS-milk containing 0.1% Tween 20, the membranes were exposed to 1:10,000 peroxidase-labelled anti-horse IgG (Sigma) for 2 h at room temperature. Unbound secondary antibodies were washed off and immunoreactive bands were visualized using diaminobenzidine (Sigma) and H_2_O_2_. The commercial antibothropic serum is produced at Butantan Institute by hyperimmunization of horses using a mixture of five *Bothrops* species venoms: *B. jararaca* (50%), *B. alternatus* (12.5%), *B. jararacussu* (12.5%), *B. moojeni* (12.5%) and *B. neuwiedi* (12.5%).

### Statistical analysis

Results are expressed as mean ± SD of triplicates. The significance of differences between the means of the venoms was determined by one-way ANOVA with Tukey as a *posteriori* test and venom pools were analyzed using Student’s *t*-test using GraphPad Prism 7.03 software, where *p* < 0.05 was considered significant.

## Results and Discussion

Differences in the composition and activity of snake venoms from the same species are a worldwide researchers concern. These differences can influence directly in the antivenom production and in the success of patient treatment [54-57].

### Compositional analysis 

Although *B. atrox* venom has been analyzed in several aspects [30,^58^-^60^], this work showed, for the first time, a comparative study of the venom extracted from female and male siblings, born in captivity and kept under controlled environmental conditions.

**Figure 1.**
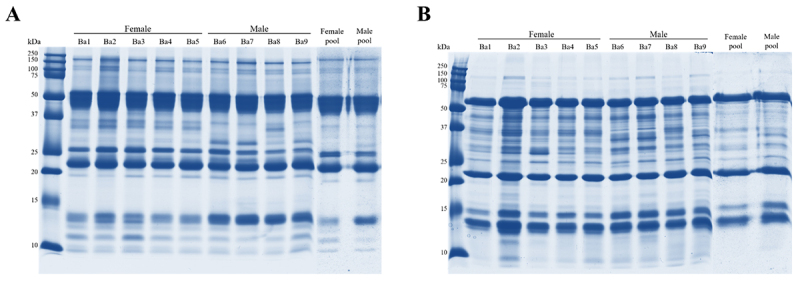
One-dimensional electrophoresis (1-DE) profile of *B. atrox* venoms under **(A)** non-reducing and **(B)** reducing conditions. Individual female (Ba1 to Ba5), male (Ba6 to Ba9) and respective pools were used and are indicated above the gel.

Electrophoretic profiles were evaluated, showing similar band patterns with few differences between individuals and pools. Individual analysis of non-reduced venoms showed a common band of ~35 kDa (Figure 1A), which is only present in the venoms of females and of Ba8 among males, and another band of ~30 kDa that is present only in the venom of males, except for Ba8. These two bands might be associated to P-II SVMP and SVSP respectively, in accordance with their molecular masses [24], and their presence and absence are reflected in the pool, although faint (especially the ~35 kDa band). Moreover, it is possible to observe bands of less intensity between 25-50 kDa (probably CRISP, GPC, P-I and P-III SVMP and SVSP) and over 100 kDa (most likely PDE). These results have been observed not only in *B. atrox* but also in other snakes of the *Bothrops* genus, and are supported by several works [31,61-63].

**Figure 2.**
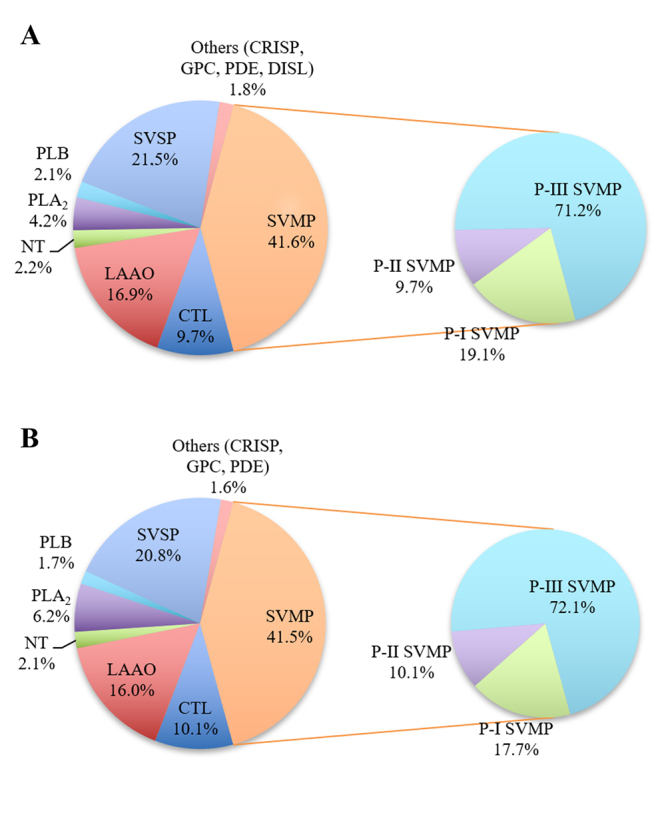
Graphical overview of toxin classes identified in *B. atrox* **(A)** female and **(B)** male venom pools by in nanoESI-qTOF. CRISP: cystein-rich secretory protein; CTL: C-type lectin; DISL: disintegrin-like protein; GPC: glutaminyl-peptide cyclotransferases; LAAO: L-amino acid oxidase; NGF: nerve growth factor; NT: nucleotidase; PDE: phosphodiesterase; PLA_2_: phospholipase A_2_; PLB: phospholipase B; SVMP: snake venom metalloproteinase; SVSP: snake venom serine protease.

In order to compare the composition of female and male *B. atrox* venoms, they were pooled according to gender and submitted to in-solution trypsin digestion followed by LC-MS/MS analysis on a Synapt G2 mass spectrometer (Waters). The results obtained allowed to identify 112 different protein compounds (Table 1 and Additional file 3), of which 105 were common proteins between female and male venom pools and 7 were unique to females. Proteins identified belong to the following families: SVMPs, SVSPs, LAAOs, CTLs, PLA_2_s, nucleotidase (NT), phospholipase B (PLB), glutaminyl-peptide cyclotransferases (GPCs), cysteine-rich secretory protein (CRISP), and disintegrin-like protein (DISL) (Figure 2, Table 1 and Additional file 3); the first five families are the main compounds in *Bothrops* venoms [32,64-66]. The unique proteins identified in the female venom were one LAAO, one P-I SVMP, one P-III SVMPs, one DISL, one CRISP, and two fragments of SVSPs. The *Bpic*-LAAO is a high weight protein of 65 kDa that causes edema and inhibition of platelet aggregation [67]; the P-I SVMP (barnettlysin-1) is non-hemorrhagic and is known to cleave many substrates, including fibrin(ogen), but not collagen [68]; VAP-1 is a P-III SVMP related to hemorrhagic activity, but is unable to cleave collagen [69]; leberagin-C is a DISL that inhibits platelet aggregation [70]; the exclusive CRISP found in the female venom was catrin-2, which weakly blocks muscle contraction induced by K^+^ and Ca^2+^ channels [71].

**Table 1.** Identification of protein compounds found in female and male *B. atrox* venom pools, by LC-MS/MS. Proteins showing statistically different abundance (fold change ≥ 1.5 or ≤ 0.67; *p* < 0.05) are bolded. The last seven proteins listed were identified exclusively in the female venom pool.

							Average intensity			
Identified protein	Organism	Protein family	Protein entry	WM (Da)	Reported peptides	Sequence coverage	Female	Male	Fold change	***p* value**
C-type lectin BiL	*Bothrops insularis*	CTL	Q6QX33	19149.44	21	86.08	13985	140107	10.0186	0.060
Thrombin-like enzyme collinein-4 (Fragments)	*Crotalus durissus collilineatus*	SVSP	C0HK18	7357.03	5	62.50	8694	64480	7.4166	0.000
L-amino-acid oxidase (Fragment)	*Bothrops jararacussu*	LAAO	Q6TGQ9	56630.92	50	68.01	5913	37126	6.2782	0.000
Protein C activator	*Agkistrodon contortrix contortrix*	SVSP	P09872	25790.23	3	11.26	898	5233	5.8284	0.028
Venom plasminogen activator LV-PA	*Lachesis muta muta*	SVSP	Q27J47	28746.44	19	57.36	14125	75746	5.3625	0.000
Zinc metalloproteinase-disintegrin-like acurhagin	*Deinagkistrodon acutus*	P-III SVMP	Q9W6M5	70766.71	38	57.87	58036	252611	4.3527	0.007
Zinc metalloproteinase/disintegrin	*Agkistrodon contortrix contortrix*	P-II SVMP	Q805F6	55166.31	11	31.47	9794	42313	4.3205	0.000
Alpha-fibrinogenase shedaoenase	*Gloydius shedaoensis*	SVSP	Q6T5L0	27100.55	10	43.28	602	2526	4.1963	0.001
Thrombin-like enzyme asperase	*Bothrops asper*	SVSP	Q072L6	28703.23	18	79.54	14337	58785	4.1003	0.001
Basic phospholipase A2 Bs-N6	*Bothriechis schlegelii*	PLA_2_	Q6EER4	16335.43	6	52.90	6758	24163	3.5754	0.004
Snake venom serine protease BthaTL	*Bothrops alternatus*	SVSP	Q6IWF1	26313.89	8	23.18	18619	61549	3.3057	0.000
C-type lectin TsL	*Trimeresurus stejnegeri*	CTL	Q9YGP1	19205.52	17	77.85	74050	224342	3.0296	0.013
Snake venom serine protease 2C	*Trimeresurus gramineus*	SVSP	O13062	28727.02	5	17.12	5156	14150	2.7443	0.000
L-amino-acid oxidase	*Gloydius halys*	LAAO	Q6STF1	57524.09	34	65.67	26729	57921	2.1670	0.017
Snaclec A8 (Fragment)	*Macrovipera lebetina*	CTL	B4XSZ8	15697.29	3	31.3	27971	51075	1.8260	0.014
Snaclec GPIB-binding protein subunit alpha	*Bothrops jararaca*	CTL	Q9PSM6	17118.95	10	61.97	35588	64211	1.8043	0.001
Snake venom serine protease KN6	*Trimeresurus stejnegeri*	SVSP	Q71QJ2	29168.04	3	28.08	11055	19401	1.7550	0.030
Zinc metalloproteinase-disintegrin-like lachestatin-1	*Lachesis muta rhombeata*	P-III SVMP	C5H5D5	48795.52	27	56.77	88267	152180	1.7241	0.201
Zinc metalloproteinase-disintegrin-like brevilysin H6	*Gloydius brevicaudus*	P-III SVMP	P0C7B0	70441.96	40	52.46	187886	323558	1.7221	0.001
Phospholipase-B 81	*Drysdalia coronoides*	PLB	F8J2D3	64445.59	9	18.63	42840	70155	1.6376	0.168
Zinc metalloproteinase-disintegrin-like halysase	*Gloydius halys*	P-III SVMP	Q8AWI5	69875.97	44	74.10	130855	210139	1.6059	0.022
Basic phospholipase A2 myotoxin III	*Bothrops asper*	PLA2	P20474	16549.88	8	48.55	367558	560726	1.5255	0.033
Snake venom serine protease catroxase-1	*Crotalus atrox*	SVSP	Q8QHK3	29309.28	7	37.40	4874	7409	1.5202	0.025
Zinc metalloproteinase-disintegrin-like 3a	*Crotalus adamanteus*	P-III SVMP	J3S830	70950.42	18	33.28	22867	34298	1.4999	0.365
Snake venom serine proteinase 12	*Crotalus adamanteus*	SVSP	J3RY93	29411.40	8	41.92	53932	77462	1.4363	0.007
Glutaminyl-peptide cyclotransferase	*Bothrops jararaca*	GPC	Q9YIB5	42489.45	12	40.49	62733	88287	1.4073	0.019
Snake venom serine proteinase 5	*Crotalus adamanteus*	SVSP	F8S116	28707.56	3	40.31	2683	3768	1.4043	0.582
Zinc metalloproteinase-disintegrin-like Eoc1	*Echis ocellatus*	P-III SVMP	Q2UXR0	70918.30	42	65.80	63008	85189	1.3520	0.005
Basic phospholipase A2 Cvv-N6	*Crotalus viridis viridis*	PLA_2_	Q71QE8	16798.19	7	55.07	139181	185224	1.3308	0.014
Zinc metalloproteinase-disintegrin-like crotastatin	*Crotalus durissus terrificus*	P-III SVMP	Q076D1	48561.06	16	46.32	5623	7374	1.3115	0.086
Snake venom metalloproteinase atroxlysin-1	*Bothrops atrox*	P-I SVMP	P85420	23317.34	20	68.32	499407	642866	1.2873	0.004
Snake venom metalloproteinase BaP1	*Bothrops asper*	P-I SVMP	P83512	46505.98	24	69.36	26950	33833	1.2554	0.687
Glutaminyl-peptide cyclotransferase	*Boiga dendrophila*	GPC	A7ISW2	42218.09	11	42.12	55102	68785	1.2483	0.056
Zinc metalloproteinase/disintegrin	*Bothrops insularis*	P-II SVMP	Q5XUW8	54567.67	31	71.85	229892	286035	1.2442	0.025
C-type lectin PAL	*Bitis arietans*	CTL	Q9PSN0	16671.42	14	91.85	89254	110591	1.2391	0.457
L-amino-acid oxidase (Fragments)	*Bothrops atrox*	LAAO	P0CC17	13700.22	12	67.23	12327	14813	1.2016	0.141
L-amino acid oxidase Bs29 (Fragment)	*Bothriechis schlegelii*	LAAO	A0A024BTN9	56775.56	26	50.00	172230	199529	1.1585	0.149
L-amino-acid oxidase (Fragment)	*Bothrops moojeni*	LAAO	Q6TGQ8	54892.89	57	77.82	625580	705521	1.1278	0.003
Snake venom metalloproteinase BjussuMP-2 (Fragment)	*Bothrops jararacussu*	P-I SVMP	Q7T1T4	42470.33	21	60.98	142041	159446	1.1225	0.014
Snake venom serine protease homolog	*Bothrops jararacussu*	SVSP	Q7T229	29338.42	18	64.23	46847	52470	1.1200	0.387
L-amino-acid oxidase	*Crotalus adamanteus*	LAAO	F8S0Z5	59166.09	36	70.35	372513	413346	1.1096	0.056
Snake venom serine protease 2	*Protobothrops flavoviridis*	SVSP	O13057	29325.57	8	35.38	60671	66267	1.0922	0.922
Snake venom serine protease CL2	*Trimeresurus stejnegeri*	SVSP	Q71QI2	28550.56	8	29.84	48626	52789	1.0856	0.941
Venom phosphodiesterase 2	*Crotalus adamanteus*	PDE	J3SBP3	93177.63	21	27.41	19296	20865	1.0813	0.083
Thrombin-like enzyme bhalternin	*Bothrops alternatus*	SVSP	P0CG03	28672.50	11	44.23	64162	68787	1.0721	0.457
Thrombin-like enzyme bilineobin	*Agkistrodon bilineatus*	SVSP	Q9PSN3	27162.92	10	41.28	139687	148613	1.0639	0.543
Snake venom serine protease BITS01A	*Bothrops insularis*	SVSP	Q8QG86	29041.26	10	28.40	119557	127189	1.0638	0.602
Cysteine-rich venom protein	*Echis coloratus*	CRISP	P0DMT4	25611.34	9	56.36	17568	18640	1.0611	0.088
Platelet-aggregating proteinase PA-BJ (Fragment)	*Bothrops jararaca*	SVSP	P81824	26426.18	5	19.41	35227	36497	1.0361	0.700
Zinc metalloproteinase-disintegrin-like	*Crotalus durissus durissus*	P-III SVMP	Q2QA02	70515.28	29	46.14	299846	304600	1.0159	0.848
L-amino-acid oxidase	*Demansia vestigiata*	LAAO	A6MFL0	59262.33	28	44.68	110123	111821	1.0154	0.826
Zinc metalloproteinase-disintegrin-like 4a	*Crotalus adamanteus*	P-III SVMP	F8S108	70077.18	47	79.51	298698	289286	0.9685	0.501
Thrombin-like enzyme gyroxin analog	*Lachesis muta muta*	SVSP	P33589	26313.56	4	37.72	782803	757006	0.9670	0.920
C-type lectin BJcuL	*Bothrops jararacussu*	CTL	P83519	19280.52	19	85.53	28257	27164	0.9613	0.498
Venom serine proteinase-like protein 2	*Macrovipera lebetina*	SVSP	Q9PT40	29577.96	9	32.69	226426	217642	0.9612	0.558
Zinc metalloproteinase-disintegrin-like agkihagin	*Deinagkistrodon acutus*	P-III SVMP	Q1PS45	69796.95	32	61.18	434004	407477	0.9389	0.248
Cysteine-rich venom protein 2	*Sistrurus catenatus edwardsii*	CRISP	B0VXV6	27397.73	4	20.08	9220	8641	0.9372	0.698
Zinc metalloproteinase-disintegrin-like BjussuMP-1 (Fragment)	*Bothrops jararacussu*	P-III SVMP	Q1PHZ4	63923.76	20	52.10	466233	434727	0.9324	0.245
Snake venom serine proteinase 14	*Crotalus adamanteus*	SVSP	J3SDW9	28098.86	10	23.32	80634	74962	0.9297	0.782
Snake venom 5'-nucleotidase	*Crotalus adamanteus*	NT	F8S0Z7	65309.44	23	43.71	271089	251012	0.9259	0.181
Snaclec GPIB-binding protein subunit beta	*Bothrops jararaca*	CTL	Q9PSM5	14697.09	5	51.22	269245	242897	0.9021	0.041
L-amino-acid oxidase	*Calloselasma rhodostoma*	LAAO	P81382	58620.22	32	65.89	220059	197551	0.8977	0.112
Zinc metalloproteinase/disintegrin	*Bothrops jararaca*	P-II SVMP	Q98SP2	54752.21	33	86.37	64025	56088	0.8760	0.473
Factor V activator RVV-V alpha	*Daboia siamensis*	SVSP	P18964	26866.45	5	26.27	30237	25481	0.8427	0.002
Thrombin-like enzyme kangshuanmei	*Gloydius brevicaudus*	SVSP	P85109	27116.53	13	66.95	5344	4461	0.8349	0.398
Zinc metalloproteinase-disintegrin-like bothropasin	*Bothrops jararaca*	P-III SVMP	O93523	70437.19	58	70.00	331793	276693	0.8339	0.046
Thrombin-like enzyme acutobin	*Deinagkistrodon acutus*	SVSP	Q9I8X2	29499.43	10	39.23	22121	17310	0.7825	0.017
Snake venom serine protease HS114	*Bothrops jararaca*	SVSP	Q5W959	28527.14	13	64.73	465717	357499	0.7676	0.005
Zinc metalloproteinase-disintegrin-like batroxstatin-3 (Fragment)	*Bothrops atrox*	P-III SVMP	C5H5D4	48223.70	22	64.25	281270	215162	0.7650	0.003
Thrombin-like enzyme 2	*Trimeresurus albolabris*	SVSP	A7LAC7	30003.48	13	37.69	159302	120556	0.7568	0.005
Snake venom metalloproteinase bothrojaractivase (Fragments)	*Bothrops jararaca*	P-I SVMP	P0C7A9	7166.29	6	83.33	98033	74020	0.7550	0.427
Neutral phospholipase A2 agkistrodotoxin	*Gloydius halys*	PLA_2_	P14421	14666.95	2	28.69	52707	39464	0.7488	0.000
Zinc metalloproteinase-disintegrin-like berythractivase	*Bothrops erythromelas*	P-III SVMP	Q8UVG0	70812.88	51	70.10	48311	35836	0.7418	0.219
Zinc metalloproteinase-disintegrin-like VAP2B	*Crotlus atrox*	P-III SVMP	Q90282	70472.21	34	45.16	145163	104879	0.7225	0.023
Snake venom metalloproteinase leucurolysin-A	*Bothrops leucurus*	P-I SVMP	P84907	23361.50	13	70.79	49037	35171	0.7172	0.090
Zinc metalloproteinase-disintegrin-like jararhagin (Fragment)	*Bothrops jararaca*	P-III SVMP	P30431	66150.04	55	79.16	673083	479418	0.7123	0.008
Zinc metalloproteinase-disintegrin-like EoVMP2	*Echis ocellatus*	P-III SVMP	Q2UXQ5	71650.55	13	25.94	174777	121426	0.6947	0.044
Snake venom 5'-nucleotidase	*Gloydius brevicaudus*	NT	B6EWW8	65118.26	19	43.88	34948	24257	0.6941	0.003
C-type Lectin CRL	*Crotalus ruber ruber*	CTL	P84987	16787.69	13	86.67	37879	25999	0.6864	0.000
L-amino-acid oxidase	*Daboia russelii*	LAAO	G8XQX1	57287.19	31	59.33	174807	119441	0.6833	0.000
Snaclec bothroinsularin subunit alpha	*Bothrops insularis*	CTL	P0C929	15608.25	5	53.03	50505	34421	0.6815	0.442
Zinc metalloproteinase/disintegrin PMMP-2	*Protobothrops mucrosquamatus*	P-II SVMP	E9NW27	55667.31	11	28.93	228990	153973	0.6724	0.072
Zinc metalloproteinase/disintegrin VMP-II	*Crotalus viridis viridis*	P-II SVMP	C9E1R9	55068.19	20	43.93	17170	11172	0.6506	0.002
Phospholipase B	*Crotalus adamanteus*	PLB	F8S101	64391.42	37	57.14	239412	153708	0.6420	0.000
Helicopsin (Fragments)	*Helicops angulatus*	CRISP	P0DJG8	2619.94	2	61.90	14992	9585	0.6393	0.047
L-amino-acid oxidase	*Echis ocellatus*	LAAO	B5U6Y8	56922.72	28	53.77	64721	40130	0.6201	0.405
Zinc metalloproteinase-disintegrin-like HV1	*Protobothrops flavoviridis*	P-III SVMP	Q90ZI3	70415.65	31	60.78	108132	65378	0.6046	0.110
Zinc metalloproteinase-disintegrin-like atrase-A	*Naja atra*	P-III SVMP	D5LMJ3	70420.96	8	22.41	120504	69488	0.5766	0.073
C-type lectin BpLec	*Bothrops pauloensis*	CTL	P86970	16736.51	24	100.00	673071	384686	0.5715	0.000
L-amino acid oxidase	*Cerastes cerastes*	LAAO	X2JCV5	58841.75	37	66.86	130921	74583	0.5697	0.529
L-amino-acid oxidase	*Trimeresurus stejnegeri*	LAAO	Q6WP39	59000.48	22	42.83	19085	10858	0.5689	0.008
L-amino-acid oxidase	*Oxyuranus scutellatus scutellatus*	LAAO	Q4JHE3	59411.80	11	25.15	72531	35505	0.4895	0.040
L-amino-acid oxidase	*Vipera ammodytes ammodytes*	LAAO	P0DI84	55090.00	24	53.72	88177	41835	0.4744	0.001
Zinc metalloproteinase-disintegrin-like HF3	*Bothrops jararaca*	P-III SVMP	Q98UF9	69862.46	26	46.53	60097	28331	0.4714	0.000
Snake venom serine protease ussurin	*Gloydius ussuriensis*	SVSP	Q8UUJ2	26885.19	11	46.19	61876	28871	0.4666	0.002
Snake venom metalloproteinase BmooMPalpha-I	*Bothrops moojeni*	P-I SVMP	P85314	23621.54	12	58.05	22457	10290	0.4582	0.007
Snake venom metalloproteinase kistomin	*Calloselasma rhodostoma*	P-I SVMP	P0CB14	48187.44	11	44.6	8736	3967	0.4541	0.417
Venom plasminogen activator	*Agkistrodon piscivorus leucostoma*	SVSP	E5L0E5	28762.35	13	59.69	124404	55540	0.4464	0.001
Zinc metalloproteinase-disintegrin-like alternagin (Fragment)	*Bothrops alternatus*	P-III SVMP	P0C6R9	21655.15	9	48.98	18356	8191	0.4462	0.002
Thrombin-like enzyme bothrombin	*Bothrops jararaca*	SVSP	P81661	26268.74	11	78.45	257720	105515	0.4094	0.001
Snaclec A6	*Macrovipera lebetina*	CTL	B4XSZ6	18222.21	3	25	26496	9530	0.3597	0.002
Venom serine proteinase-like protein 1	*Bitis gabonica*	SVSP	Q6T6S7	29666.79	7	21.92	20605	5869	0.2848	0.050
Cysteine-rich venom protein triflin	*Protobothrops flavoviridis*	CRISP	Q8JI39	27664.76	15	62.92	36767	10146	0.2760	0.219
L-amino acid oxidase Lm29	*Lachesis muta*	LAAO	J7H670	58931.49	30	67.25	198453	27806	0.1401	0.000
Thrombin-like enzyme halystase	*Gloydius blomhoffii*	SVSP	P81176	27167.66	9	39.08	37809	5117	0.1353	0.002
L-amino acid oxidase (Fragment)	*Bothrops pictus*	LAAO	X2L4E2	56747.89	42	59.44	10767	-	-	-
Zinc metalloproteinase barnettlysin-1	*Bothrops barnetti*	P-I SVMP	P86976	21322.16	12	71.78	238832	-	-	-
Zinc metalloproteinase-disintegrin-like VAP1	*Crotalus atrox*	P-III SVMP	Q9DGB9	70127.33	27	65.08	13072	-	-	-
Cysteine-rich venom protein catrin	*Crotalus atrox*	CRISP	Q7ZT99	27558.99	7	37.08	28648	-	-	-
Beta-fibrinogenase brevinase	*Gloydius blomhoffii*	SVSP	Q9PT51	26409.51	3	12.02	310	-	-	-
Disintegrin-like leberagin-C	*Macrovipera lebetina transmediterranea*	DISL	C0LZJ5	24231.26	2	13.17	4196	-	-	-
Snake venom serine protease rhinocerase (Fragments)	*Bitis rhinoceros*	SVSP	P86497	10262.82	2	56.18	164	-	-	-

CRISP: cysteine-rich secretory protein; CTL: C-type lectin; DISL: disintegrin-like protein; GPC: glutaminyl-peptide cyclotransferases; LAAO: L-amino acid oxidase; NGF: nerve growth factor; NT: nucleotidase; PDE: phosphodiesterase; PLA_2_: phospholipase A_2_; PLB: phospholipase B; SVMP: snake venom metalloproteinase; SVSP: snake venom serine protease.

Sousa et al. [30] examined the venom composition of *B. atrox* according to their habitats and the proteomics analyses showed some differences in comparison to our study, such as the presence of hyaluronidases, which were not identified in this work. It is interesting to note that the relative percentages of LAAOs and SVSPs obtained by our group by MS analysis were higher than the aforementioned study, 16% in comparison to ~9% for LAAOs, and 21% in comparison to 10% to 14% for SVSPs, respectively. Another study indicates higher percentages of SVMPs than found here and have not detected any PLB [60].

### Functional analysis

**Figure 3.**
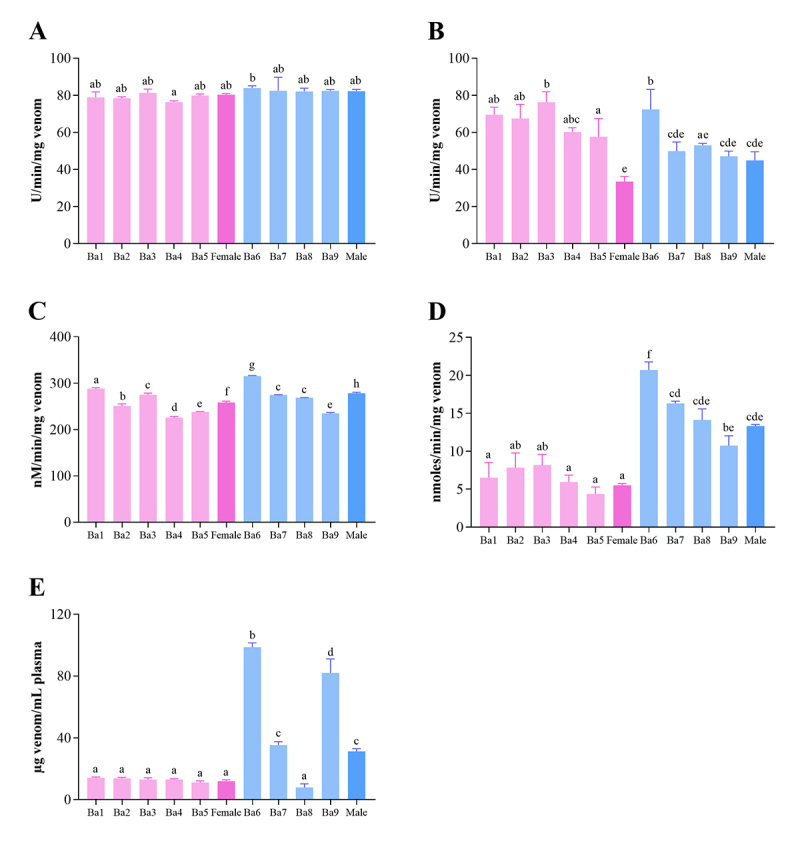
Enzymatic activities of *B. atrox* venom (individual and pool). The data were expressed as mean ± SD, n = 3. Different letters indicate statistical difference (ANOVA, *p* < 0.05). **(A)** Caseinolytic activity; **(B)** collagenolytic activity; **(C)** LAAO activity; **(D)** PLA_2_ activity; **(E)** MCD.

Proteolytic activities over casein and collagen did not show statistical difference between female and male pools, although some individual variations were observed. For caseinolytic activity (Figure 3A), only Ba4 and Ba6 showed statistical difference. As for collagenolytic activity (Figure 3B), individual variability was more evident. Caseinolytic activity may be associated with SVMP and SVSP, since casein is a substrate degraded by these families of proteins [72,73] and, in this study, neither of these two protein families differed between the pools analyzed by MS (Figure 2).

LAAOs have the ability to induce or inhibit platelet aggregation, in addition to promoting hemorrhage, hemolysis, the appearance of edema, and other biological activities [74-76]. The percentage of LAAOs found in female venom pool analyzed by MS was slightly higher than for males. However, male venom pool showed higher activity compared to the female pool (Figure 3C). Although contrasting, the same behavior was observed in *B. moojeni* [34]. Similar to collagenolytic activity, LAAO activity differed individually.

PLA_2_ activity (Figure 3D) of *B. atrox* venom showed a strong individual variation, but, overall, the venom of males presented higher activity than female venoms. This was also reflected in the pools: male pool had a higher activity than female pool. Similar results were also observed in other species, like *B. jararaca* and *B. moojeni* [34,77]. This result was corroborated by mass spectrometry identification, in which a higher percentage of PLA_2_ was found in the male pool. In Viperidae, the PLA_2_s found in snake venoms have been divided into two groups: with catalytic activity (Asp49 - D49) and without catalytic activity (Lys49 - K49). The substitution of the amino acid residue Asp-49 for Lys-49 consequently causes loss of calcium binding, primordial for its enzymatic activity [78].

In MCD analysis (Figure 3E), female venoms showed very similar activity among them, as well as the pool. As for males, Ba8 showed the highest activity, comparable to females, while the others presented much lower activity in comparison to females. The MCD is most likely attributed to procoagulant SVMPs and SVSPs, relating to activation of prothrombin and factor X of the clotting cascade [79,80]. Despite similarities in abundance between the groups, the female pool showed, altogether, slightly more SVSP than male pool in proteomic analysis. Besides, female venom pool had slightly higher amount of thrombin-like than the male pool (11.0% and 10.6%, respectively) (Figure 2, Table 1 and Additional file 3). Also, if we consider that 112 proteins were identified in the mass spectrometry of *B. atrox* snake venoms used in this study and that each protein-protein interaction responds differently depending on the compounds involved [16,17], this difference may also be attributed to the synergy between protein families in local and systemic damage. It is important to highlight the limitations of the use of plasma without recalcification in this work because this may influence the time of clotting of each venom. Although it is known that SVMPs from the group A are not dependant of cofactors (including calcium) to activate prothrombin [81], a recent study [82] showed that the procoagulant effects of *Bothrops* genus snake venoms are highly dependant of calcium and that the dependency varies between populations. Although the results obtained herein show that, in the absence of calcium, the venom of females *B. atrox* is prone to be more coagulant, it is important to consider the role of calcium upon snake venom coagulopaties, even for independent calcium prothrombin activators [83], which may result in a misinterpretation of the relative toxicities.

Individual differences were observed in enzymatic activities, highlighting the importance of individual analysis when possible. Despite some individual differences, a pattern between the activities of females and males can be correlated, so, for *in vivo* tests, the pool was chosen for analysis. Galizio et al. [84] reinforce the importance of the individual analysis, but for ethical issues pools were used to reduce the number of animals utilized in the *in vivo* experiments.

MHD of male venoms was lower when compared to females (2.7 and 4.8 μg/animal, respectively), indicating that female venom pool needs more than 43.8% of venom to generate the corresponding hemorrhagic halo to MHD, than male venom pool. Saldarriaga et al. [51] found 1.8 µg/animal as MHD for adult (3 years old) *B. atrox*, a minor dose than the one found in this work. Although considered adults, these snakes were younger than the ones in our work. Guércio et al. [24] analyzed the ontogenetic variation in the proteome of *B. atrox* and identified more P-III SVMPs in younger snakes than in adults, which could explain the higher hemorrhagic effects observed elsewhere [51]. The difference in MHD observed between female and male pools in our work may be attributed to the different abundance of P-III SVMPs identified in the venom pools.

LD_50_ of female venom pool of *B. atrox* (104.3 µg/animal; CI: 73.3−151.2 µg/animal) was slightly lower than that of the male (118.4 µg/animal; CI: 87.2−164.8 µg/animal), but with no statistical difference. Although differences were observed in some activities, this is not reflected in the venom lethality. Saldarriaga et al. [47] found 81.4 µg/mice as LD_50_ for adult *B. atrox*, a minor dose than found in this work. Also, Sousa et al. [30] compared the geographic variation of *B. atrox* and reported a lower LD_50_ than herein observed and suggested a correlation with the lower hemorrhagic activity. This is consistent with the results of the procoagulant and hemorrhagic activities, which are apparently related to the lethality of the venom [85,86]. Another study relates a lack of hemorrhagic activity associated with a higher lethality in *Daboia russelii* [87].

There was a marked difference between hemorrhagic and procoagulant activities between the venom of males and females, and these results may relate with the metabolic requirements of each sex. The metabolic rate of males and females is different, and it has been previously shown in viperids that females have a higher oxygen consumption, which is related to the animal's mass [88]. Since *B. atrox* is a species displaying sexual dimorphism, in which females are usually larger than males, it is possible that females have a higher energy demand due to their larger size, in addition to the need of extra energy reserved for reproduction [89].

Regarding MHD, the variation may have been caused by the relative abundance of proteins with hemorrhagic activity, which is slightly lower in the female venom pool than in the male venom pool. This activity may be under the influence of other proteins and/or the synergistic effect of other compounds in the venom.

### Immunorecognition by antibothropic serum

**Figure 4.**
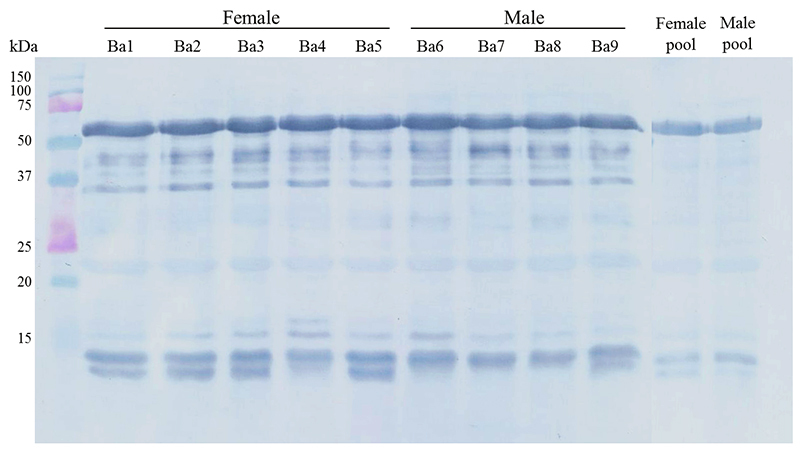
Immune interaction between the proteins of *B. atrox* venoms and the antibothropic serum by western blotting. Individual female (Ba1 to Ba5), male (Ba6 to Ba9) and respective pools were used and are indicated above the gel.

The antivenom produced at Butantan Institute is composed by antibodies raised in horses, using a mixture of *B. jararaca* (50%), *B. jararacussu* (12.5%), *B. alternatus* (12.5%), *B. moojeni* (12.5%) and *B. neuwiedi* complex (12.5%) venom. Although *B. atrox* is not included in the venom pool used to produce the antivenom, it seems to have a moderate reaction with the serum (Figure 4).

Overall, the antibothropic serum produced at Butantan Institute recognized all venoms similarly, especially the ones with higher and lower molecular weights (Figure 4). Curiously, the band between 20 and 25 kDa were not well recognized by the serum in all groups, although it’s very strong in the gel (Figure 1B). Analyzing the MS (Table 1 and Additional file 3), it is concluded that this band probably represents a PI-SVMP. Other studies concerning *B. atrox* venom that also tested the immunerecognition using the antibothropic serum produced at Butantan Institute, showed that this reaction is not as strong as with other species’ venom; and geographic variation seems to have great influence in the reactivity of the venoms to the antivenom [51,58,62,90]. Moreover, Sousa and colleagues [30] found striking differences in the neutralization of *in vivo* activities of *B. atrox* venoms from different habitats, regardless of the similarity in the reaction observed by ELISA.

## Conclusion

Several studies have shown that *B. atrox* venom may have variability in their biological activity and protein composition. This work extends the outlook regarding this variability, showing that female and male venoms of *B. atrox* siblings, under the same controlled environmental conditions, present subtle differences in their composition and activities. Moreover, it was observed individual variability in the characteristics of venoms, indicating that, in addition to aspects such as, geographical location, ontogeny, sex and diet, there are several unknown factors that result in the venom plasticity and physiological effects.

### Abbreviations

1-DE: one dimensional electrophoresis; ADH: alcohol dehydrogenase; ANOVA: analysis of variance; BSA: bovine serum albumin; CEUAIB: Comissão de Ética no Uso de Animais do Instituto Butantan (Ethical Committee for the Use of Animals of Butantan Institute); CI: confidence interval; CONCEA: Conselho Nacional de Controle de Experimentação Animal (Brazilian Council of Animal Experimentation Control); CRISP: cysteine-rich secretory protein; CTL: C-type lectin; DISL: disintegrin-like protein; DTT: dithiothreitol; GPC: glutaminyl-peptide cyclotransferases; IAA: iodoacetamide; kDa: kilodalton; LAAO: L-amino acid oxidase; LC-MS/MS: liquid chromatography-mass spectrometry/mass spectrometry; LD_50_: lethal dose 50%; MCD: minimum coagulant dose; MHD: minimum hemorrhagic dose; NOBA: 4-nitro-3-octanoyloxy-benzoic acid; NT: nucleotidase; PDE: phosphodiesterase; PLA_2_: phospholipase A_2_; PLB: phospholipase B; PVDF: polyvinylidene difluoride; RP-HPLC: reverse-phase high performance liquid chromatography; RP-UPLC: reverse-phase ultra performance liquid chromatography; SD: standard deviation; SVMP: snake venom metalloproteinase; SVSP: snake venom serine proteinase; TBS: Tris-buffered-saline; TCA: trichloroacetic acid; TFA: trifluoroacetic acid.

## Supplementary material

The following online material is available for this article:

Additional file 1. Individual information of snakes used in this work.

Additional file 2. Data of the processed spectra.

Additional file 3. Protein identification by LC-MS/MS.
