# Supplementary material for: Venom complexity of Bothrops atrox (common lancehead) siblings
Source: J Venom Anim Toxins Incl Trop Dis. 2020 Oct 12;26:e20200018. doi: 10.1590/1678-9199-JVATITD-2020-0018 (PMC7553035; doi:10.1590/1678-9199-JVATITD-2020-0018)
Supplement: Additional file 1. [file 1678-9199-jvatitd-26-e20200018-s1.pdf]

J Venom Anim Toxins incl Trop Dis
https://doi.org/10.1590/1678-9199-JVATITD-2020-0018

Supplementary Material to "Venom complexity of *Bothrops atrox* (common lancehead) siblings"

Additional file 1. Individual information of snakes used in this work.

ID	Sex	Weight (g)	Size (cm)	Venom color
Ba1	♀	1,6660	130	Yellow
Ba2	♀	845	114.5	Yellow
Ba3	♀	1,365	145	Yellow
Ba4	♀	1,445	136	Yellow
Ba5	♀	1,695	142	Yellow
Ba6	♂	510	102	Yellow
Ba7	♂	805	119.5	Yellow
Ba8	♂	860	120	Yellow
Ba9	♂	685	119	Yellow
