# Supplementary material for: Venom complexity of Bothrops atrox (common lancehead) siblings
Source: J Venom Anim Toxins Incl Trop Dis. 2020 Oct 12;26:e20200018. doi: 10.1590/1678-9199-JVATITD-2020-0018 (PMC7553035; doi:10.1590/1678-9199-JVATITD-2020-0018)
Supplement: Additional file 2. [file 1678-9199-jvatitd-26-e20200018-s2.zip › Additional file 2_Legend.pdf]

J Venom Anim Toxins incl Trop Dis
https://doi.org/10.1590/1678-9199-JVATITD-2020-0018

Supplementary Material to "Venom complexity of *Bothrops atrox* (common lancehead) siblings"
